# Perrault syndrome type 3 caused by diverse molecular defects in CLPP

**DOI:** 10.1038/s41598-018-30311-1

**Published:** 2018-08-27

**Authors:** Erica J. Brodie, Hanmiao Zhan, Tamanna Saiyed, Kaye N. Truscott, David A. Dougan

**Affiliations:** 1 Department of Biochemistry and Genetics, La Trobe Institute for Molecular Science, La Trobe University, Melbourne, 3086 Victoria Australia; 2 Present Address: Department of Immunology and Pathology, Monash University, Melbourne, 3004 Victoria Australia

## Abstract

The maintenance of mitochondrial protein homeostasis (proteostasis) is crucial for correct cellular function. Recently, several mutations in the mitochondrial protease CLPP have been identified in patients with Perrault syndrome 3 (PRLTS3). These mutations can be arranged into two groups, those that cluster near the docking site (hydrophobic pocket, Hp) for the cognate unfoldase CLPX (i.e. T145P and C147S) and those that are adjacent to the active site of the peptidase (i.e. Y229D). Here we report the biochemical consequence of mutations in both regions. The Y229D mutant not only inhibited CLPP-peptidase activity, but unexpectedly also prevented CLPX-docking, thereby blocking the turnover of both peptide and protein substrates. In contrast, Hp mutations cause a range of biochemical defects in CLPP, from no observable change to CLPP activity for the C147S mutant, to dramatic disruption of most activities for the “gain-of-function” mutant T145P - including loss of oligomeric assembly and enhanced peptidase activity.

## Introduction

Perrault syndrome (PRLTS) is a rare autosomal recessive disorder that is characterised by ovarian dysgenesis in females and sensorineural hearing loss (SNHL) in both genders^1^. The disease is clinically heterogeneous and in severe cases, additional symptoms may include ataxia, neuropathies and intellectual disability. To date, mutations in six different genes have been linked to the disease: PRLTS1 is caused by compound heterozygous mutations in *HSD17B4* (17-beta hydroxysteroid dehydrogenase); PRLTS2 results from compound heterozygous mutations in *HARS2* (mitochondrial histidyl-tRNA synthetase); PRLTS3 is caused by homozygous or compound heterozygous mutations in *CLPP* (mitochondrial protease); PRLTS4 results from homozygous or compound heterozygous mutations in *LARS2* (mitochondrial leucyl-tRNA synthetase); PRLTS5 is caused by homozygous or compound heterozygous *C10orf2* (mitochondrial DNA helicase Twinkle) and PRLTS6 has been recently linked to a missense mutation in *ERAL1* (mitochondrial chaperone required for mitochondrial ribosome assembly). Most of the causative genes for PRLTS (including *CLPP*) are implicated in the maintenance of proteostasis in mitochondria, in particular mitochondrial protein translation^1–9^. Consistent with an important role for CLPP in Perrault syndrome, *CLPP* null mice (*CLPP*^−/−^) also display infertility, deafness and growth retardation^10^.

In humans, CLPP is a nuclear encoded mitochondrial protease that is directed to the mitochondrial matrix through an N-terminal targeting sequence^11^. Following import and processing, mature CLPP is proposed to assemble, initially into a ring-shaped heptamer^12^ and finally, in the presence of its cognate ATP-dependent unfoldase – CLPX, into an active barrel-shaped tetradecamer^13^. Based on studies with bacterial ClpXP homologs, formation of the human CLPXP complex is mediated by two sets of interactions, a set of *static* interactions between a loop on ClpX, containing the conserved tripeptide motif ([L/I/V]-G-[F/L]), and the hydrophobic pocket (Hp) on ClpP^14–16^, as well as a set of *dynamic* interactions between the flexible N-terminal loop of ClpP and the pore-2 loop of ClpX^14,16–18^.

Perrault syndrome type 3 (PRLTS3) is caused by mutations in *CLPP*, and to date seven different mutations have been identified in patients. Structurally, these mutations can be broadly classified into two regions. The first group of mutations (P142L, C144R, T145P, C147S and G162S) is located in, or around, the Hp of CLPP^2,4,19^. As this region is responsible for interaction with the ATPase component^2,4,16^, these mutations are proposed to impair CLPX interaction and as a consequence, CLPX-mediated proteolytic activity, but not CLPP peptidase activity^2,4^. The second group of mutations (Y229D and I208M) is located in close proximity to the catalytic triad of CLPP, and as such these mutations are proposed to alter the peptidase activity of CLPP, but not its docking to CLPX^20,21^. To date, however, neither the structural nor functional consequences of these mutations in CLPP have been extensively examined. To understand the effect of these different types of mutations, we chose to study two mutations from the first group (namely T145P and C147S, both of which were identified in the initial study by Newman and colleagues^4^) and one mutation from the second group (namely Y229D). Here we show, that each of the different CLPP mutations exhibit a specific defect, from protein assembly to peptidase activity, or their interaction with CLPX. Interestingly, of the two Hp mutants tested, only one (T145P) exhibited severely compromised activity. Surprisingly, the other mutation (C147S) had no measurable defect on CLPP function *in vitro*. In contrast to C147S, the catalytic-region mutant (Y229D) exhibited a number of significant defects in CLPP function.

## Materials and Methods

### Cloning

Constructs encoding mutant CLPP (T145P, C147S, Y229D, ∆C, RA or ECRC) were generated by site-directed mutagenesis^22^ using either *pHUE/CLPP* or *pOTB7/CLPP*^23^ as template DNA. Refer to supplementary material for primer sequences and the final plasmid constructs generated.

### Cell culture, preparation of mitochondria and *in vitro* import of radiolabelled preproteins into mammalian mitochondria

HeLa (ATCC® CCL2™) cells were grown and maintained at 70–90% confluency at 37 °C with 5% (v/v) CO_2_ in Dulbecco’s Modified Eagle Medium (DMEM; ThermoFisher Scientific) supplemented with 10% (v/v) Fetal Bovine Serum (FBS) for a maximum of one month. Mitochondria were isolated from HeLa cells as described^24^. *In vitro* import reactions were performed essentially as described^25^. Wild type and mutant precursor CLPP (preCLPP) proteins were radiolabelled in the presence of 11 µCi [^35^S]Met/Cys EXPRE^35^S ^35^S Protein Labelling Mix (specific activity >1000 Ci/mmol) (Perkin Elmer, Waltham, MA, USA), using the TnT® SP6 Coupled Reticulocyte Lysate System (Promega, Australia), according to the manufacturer’s instructions. Following import, the proteins were separated by SDS-PAGE (see below), gels were then dried, and the radiolabelled proteins visualised by digital autoradiography using a Typhoon Trio Molecular Imager (GE Healthcare).

### Protein expression and purification

With the exception of CLPP^T145P^, all recombinant proteins were expressed in BL21-Codon Plus (DE3)-RIL *Escherichia coli* cells. CLPP^T145P^ was expressed in ∆*dnaK* (EN2) *E*. *coli* cells^26^. Wild type and mutant, mature human CLPP (m-CLPP) was expressed as a His_6_Ubiquitin (H_6_Ub) fusion protein and subsequently purified by immobilised metal ion affinity chromatography (IMAC) using Ni-NTA agarose beads (QIAGEN). The H_6_Ub moiety was cleaved from the fusion protein using the deubiquitinating (DUB) enzyme, Usp2cc^27^ and the untagged protein was purified, essentially as described^28^. As required, recombinant proteins were applied to a Superdex 200 HiLoad 16/60 pg column (GE Healthcare) pre-equilibrated in GF buffer (50 mM Tris-HCl [pH 7.5], 100 mM NaCl, 200 mM KCl, 5% (v/v) glycerol, 0.025% (v/v) Triton X-100 and 20 mM MgCl_2_) in the presence or absence of 0.5 mM TCEP, connected to a NGC^TM^ Quest Plus Chromatography System (Bio-Rad) with Chromlab software v3.1.0.06. The elution profile was monitored by absorbance at 280 nm (A_280_) and the column was calibrated according to the manufacturer’s instructions using HMW Gel Filtration Calibration Kit (GE Healthcare). Mature human CLPX was expressed as a C-terminal His_10_ fusion protein and purified as described previously^23^. *E*. *coli* ClpP (EcClpP) and His_6_GFP-SsrA were expressed and purified as described^29^, while *E*. *coli* ClpX (EcClpX) was purified as described^30^. Fluorescein isothiocyanate (FITC)-casein and N-Suc-Leu-Tyr-7-amino-4-methylcoumarin (Suc-LY-amc) were purchased from Sigma-Aldrich.

### *In vitro* degradation assays

Human CLPX(P)-mediated degradation assays were performed in hXP buffer (50 mM Tris-HCl [pH 7.5], 100 mM KCl, 100 mM NaCl, 20 mM MgCl_2_, 10% (v/v) glycerol, 0.025% (v/v) Triton X-100), in the presence or absence of 1 mM DTT (as indicated). All degradation assays were performed at 30 °C in black 96 well plates (Corning flat bottom) using a SpectraMax M5e plate reader (Molecular Devices). For the degradation of FITC-casein, the final protein concentrations were as follows, 0.3 µM FITC-casein, 2.4 µM CLPX and 5.6 µM CLPP (wild type or mutant). FITC fluorescence was excited at 490 nm and the emission was monitored at 520 nm. The degradation reaction was initiated with the addition of ATP (5 mM). To determine the rate of FITC-casein degradation, the fluorescence intensity of FITC-casein (alone) was subtracted from the fluorescence intensity of FITC-casein (in the presence of the CLPX). FITC-casein protein turnover was also monitored by SDS-PAGE and visualised using a Typhoon Trio Molecular Imager as described previously^31^. For the degradation of GFP-SsrA the final concentrations were 1.2 µM ecClpX, 2.8 µM CLPP (wild type or mutant) and 1 µM GFP-SsrA. The degradation reaction was initiated with the addition of ATP (5 mM). GFP fluorescence was excited at 410 nm and the emission was monitored at 500 nm. For the CLPP-mediated degradation of Suc-LY-amc the final concentrations were as follows, 1 mM Suc-LY-amc and 5.6 µM CLPP (wild type or mutant). In this case, substrate turnover was initiated with the addition of Suc-LY-amc. Fluorescence was monitored at 460 nm (excitation at 380 nm).

### Protein separation and analysis

To identify the N-terminus of mature human CLPP, preCLPP_FLAG_ was expressed in HeLa cells, mitochondria were isolated as described^24^, then solubilised in pre-chilled mitochondrial immunoprecipitation (mito IP) buffer (50 mM Tris-HCl [pH 7.5], 100 mM KCl, 10 mM Mg-acetate, 5% (v/v) glycerol, 1% (v/v) Triton X-100) supplemented with 1 mM phenylmethylsulfonyl fluoride (PMSF). Following incubation on ice (30 min), the insoluble material was separated by centrifugation (16060 *g*, 10 min, 4 °C) and CLPP_FLAG_ recovered from the clarified lysate by immunoprecipitation. Briefly, the clarified lysate was incubated end-over-end (1 h, 4 °C) with 50 μl (settled volume) of anti-FLAG M2 affinity beads (Sigma-Aldrich) pre-equilibrated with Tris-buffered saline (TBS; 50 mM Tris-HCl [pH 7.5], 150 mM NaCl). The beads were washed (20 BV of TBS) prior to elution of the bound protein using 2 BV of 50 mM glycine-HCl [pH 3.0]. Following neutralisation (using 1 M Tris-HCl [pH 8.0]), the protein sample was separated by SDS-PAGE and then transferred to PVDF membrane using CAPS buffer (10 mM CAPS-KOH [pH 11.0], 10% (v/v) methanol) and the band of interest was excised before being subjected to seven cycles of automated Edman degradation using an Applied Biosystems 494 Procise Protein sequencing system. Sequencing and analysis was performed by the Australian Proteome Analysis Facility.

To monitor the assembly of human CLPP, recombinant wild type or mutant CLPP (4 µg) was prepared in Native-PAGE buffer (10 mM Tris-HCl [pH 7.0], 10 mM NaCl, 5 mM MgCl_2_, 10 mM KCl, 5% (v/v) glycerol), supplemented with 50 mM Bis-Tris, 50 mM NaCl, 10% (v/v) glycerol, 0.001% (w/v) PonceauS then separated using 4–16% Native-PAGE Novex Bis-Tris gels (Invitrogen) according to the manufacturer’s instructions and visualised by staining with CBB. Native HMW calibration kit (GE Healthcare) was used as molecular weight markers. To monitor the import of radiolabelled CLPP (in HeLa mitochondria), mitochondrial proteins were separated using 12.5% SDS-PAGE. Following electrophoresis, the gels were dried and then scanned by digital autoradiography using a Typhoon Trio Molecular Imager (GE Healthcare). Images were analysed using the ImageQuant 5.1 software (GE Healthcare).

### Analysis of human CLPX-CLPP complexes by co-immunoprecipitation (coIP)

To assess human CLPX-CLPP complex formation, wild type or mutant CLPP (0.5 µM) was incubated in the absence or presence of human CLPX (1 µM) in IP buffer (50 mM Tris-HCl [pH 7.5], 100 mM NaCl, 100 mM KCl, 40 mM Mg-Acetate, 10% (v/v) glycerol and 0.1% (v/v) Triton X-100), supplemented with 2 mM ATPγS (as indicated). Following a short preincubation of the proteins at room temperature, the samples were then incubated with Protein A-Sepharose (Sigma-Aldrich) containing pre-bound anti-CLPX antibodies and mixed end-over-end for 60 min at 4 °C. Following removal of the unbound proteins, the Protein A-Sepharose beads were washed, essentially as described^32^, using ice cold IP buffer supplemented with 10 mM ATP. Finally, the bound proteins were eluted with 50 mM glycine-HCl [pH 2.5]. All proteins were separated by 16.5% Tricine SDS-PAGE^33^, transferred to PVDF membrane and detected by immunodecoration with specific antisera (either anti-CLPP (Origene OTI1D3) or anti-CLPX, as described^23^).

## Results

### All PRLTS3-causing CLPP mutants are imported into mitochondria

**Figure 1:**
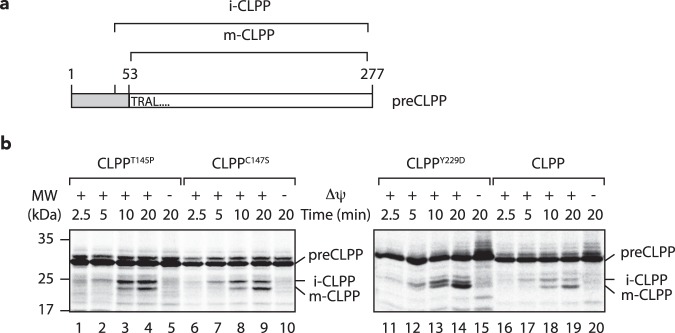
Import and processing of PRLTS3-causing mutations in CLPP. (**a**) Cartoon of Human precursor CLPP (preCLPP), illustrating the relative location of intermediate CLPP (i-CLPP) and mature CLPP (m-CLPP), including the identity of the N-terminus of m-CLPP as identified by N-terminal sequencing. (**b**) Radiolabelled precursor protein of CLPP^T145P^ (lanes 1–5), CLPP^C147S^ (lanes 6–10), CLPP^Y229D^ (lanes 11–15) and wild type CLPP (lane 16–20) was imported into mitochondria isolated from HeLa cells, in the presence or absence of a membrane potential (∆ψ) as indicated. The precursor (pre) protein was processed into an intermediate (i-) and finally mature (m-) CLPP. All radiolabelled proteins were separated by 12.5% SDS-PAGE and visualised by digital autoradiography. The full-length images are presented in Supplementary Fig. S7.

To better understand the molecular basis of the PRLTS3-causing mutations in CLPP, we introduced specific point mutations (T145P, C147S and Y229D) into human CLPP and compared their import and processing. Initially, we established the conditions for *in vitro* import of wild type CLPP into mitochondria isolated from HeLa cells (Fig. 1). As expected for a matrix located protein, the radiolabelled preprotein (preCLPP) was imported into mammalian mitochondria as determined by the time and membrane potential dependent accumulation of the processed protein (Fig. 1b, compare lanes 19 and 20). Interestingly, processing of CLPP appeared to occur in two steps, via an intermediate (here referred to as i-CLPP, Fig. 1a). We propose that the first processing step results in removal of the N-terminal presequence (likely via the matrix processing protease (MPP)) to generate the intermediate (i-CLPP), which is further processed into the mature protein (m-CLPP) via the autocatalytic removal of the CLPP propeptide. Although the final processing step is consistent with the autocatalytic processing of many ClpP proteins, including *E*. *coli* ClpP^34^ we cannot exclude that processing of i-CLPP to m-CLPP is mediated by another mitochondrial peptidase. Next, we examined the import and processing of the different PRLTS3-causing CLPP mutants. Each mutant was imported into isolated mitochondria with similar kinetics to wild type CLPP, as can be seen by the appearance of i-CLPP by ~5 min (Fig. 1b, compare lane 17 with lanes 2, 7 and 12). This was followed by the appearance of mature CLPP (m-CLPP) (Fig. 1b, compare lane 18 with lanes 3, 8 and 13). Surprisingly, although CLPP^Y229D^ was imported into mitochondria, as demonstrated by the requirement of polarised mitochondria (Fig. 1b, compare lanes 14 and 15), the processing of this mutant was different to the other proteins (wild type and the other mutants) as judged by the altered molecular weight of i-CLPP^Y229D^, suggesting that CLPP^Y229D^ is processed via an alternate mechanism (Fig. 1b, compare lanes 14 and 19). Importantly, the molecular weight of m-CLPP is seemingly unchanged for all forms of CLPP (wild type and mutant). Irrespective of whether CLPP processing occurs via slightly different pathways, we wanted to ensure that all subsequent *in vitro* experiments were performed using the fully processed form of the protein. Thus, we determined the N-terminus of wild type human CLPP via N-terminal sequencing (Edman degradation). For this purpose, we overexpressed human preCLPP bearing a C-terminal FLAG-tag (CLPP_FLAG_) in HeLa cells, isolated the tagged processed protein via immunoprecipitation (IP) from purified HeLa mitochondria, and determined the N-terminal sequence by performing seven rounds of Edman degradation. From the data obtained, we identified Thr53 as the most abundant N-terminal residue of mature human CLPP_FLAG_ (Fig. 1a). Significantly, cleavage at this site (Thr53) is not consistent with the R-2 rule of processing by the mitochondrial processing peptidase^35,36^, however it is consistent with autocatalytic propeptide cleavage of human CLPP proposed by Maurizi and colleagues^37^. It is also consistent with propeptide processing sites identified in several bacterial ClpP homologs^34,38^. Consistent with our identification of Thr53 as the N-terminal residue of mature CLPP, radiolabelled m-CLPP expressed *in organello* co-migrated with purified untagged wild type recombinant CLPP (CLPP_53–277_) used in this study (Fig. S1).

### Human CLPP forms a double-ringed complex in the absence of CLPX

**Figure 2:**
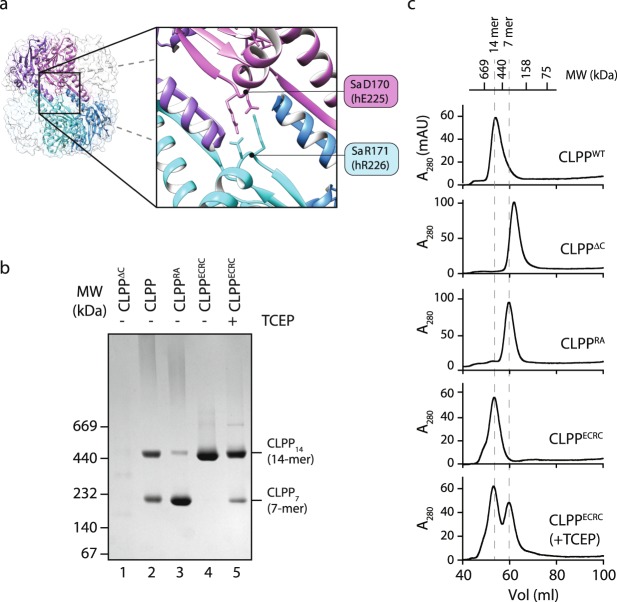
Wild type CLPP forms heptamers and tetradecamers *in vitro* in the absence of human CLPX. (**a**) Model of *Staphylococcus aureus* ClpP_14_ (SaClpP_14_) PDB: 5C90 highlighting the position of D170 and R171, equivalent to E225 and R226 in human CLPP (here termed hE225 and hR226 respectively). (**b**) Assembly of wild type or mutant CLPP under native conditions. Recombinant, untagged wild type or mutant human CLPP (4 µg) was separated by Native-PAGE and visualised by staining with Coomassie Brilliant Blue (CBB) R250. The tetradecamer (CLPP_14_) and heptamer (CLPP_7_) are indicated. The oligomeric composition of wild type human CLPP (lane 2) was compared to either CLPP^ΔC^ (lane 1), CLPP^RA^ (lane 3) or CLPP^ECRC^ either in the absence (lane 4) or presence (lane 5) of TCEP. (**c**) The tetradecamer (14-mer) and heptamer (7-mer) of recombinant, untagged wild type or mutant CLPP was separated by size exclusion chromatography (SEC) using a Superdex 200 HiLoad 16/60 pg column. Elution profiles of wild type CLPP (top panel), CLPP^∆C^ (second panel), CLPP^RA^ (middle panel), CLPP^ECRC^ in the absence (fourth panel) or presence of TCEP (bottom panel) were measured at 280 nm (A_280_). Lines indicate the peak elution volume of thyroglobulin (669 kDa), ferritin (440 kDa), aldolase (158 kDa) and conalbumin (75 kDa).

Given each mutant protein was imported into mitochondria, we wanted to examine the ability of each protein to form a functional oligomer. Initially, we used Native-PAGE to examine the oligomeric assembly of wild type human CLPP *in vitro* (Fig. 2). This technique was recently used by Osiewacz and colleagues^39^ to monitor the oligomeric state of ClpP, and consistent with their findings, we observed two oligomeric species for human CLPP (Fig. 2b, lane 2). Based on the apparent molecular weight of these species (~220 kDa and ~460 kDa), they are expected to represent the heptamer and tetradecamer of CLPP respectively. However, given that human CLPP was proposed to only form a tetradecamer in the presence of its partner protein CLPX^13^, we wanted to confirm the identity of the oligomeric species observed in Native-PAGE before examining the effect of the different PRLTS3-causing mutations on CLPP assembly. To do so, we designed three mutations in human CLPP. The first two mutations were based on the identification of an arginine sensor (Arg171) in *Staphylococcus aureus* ClpP (*Sa*ClpP), which forms a salt-bridge with Asp170 across the ring-ring interface^40^, facilitating tetradecamer formation (Fig. 2a). The first mutation in human CLPP (namely Arg226Ala herein referred to as CLPP^RA^; equivalent to the Arg171Ala mutation in *Sa*ClpP) was designed to impair tetradecamer formation. The second mutant protein (herein referred to as CLPP^ECRC^) contained two complementary point mutations at the inter-ring interface (namely Glu225Cys [equivalent to Asp170 in *Sa*ClpP] and Arg226Cys [equivalent to Arg171 in *Sa*ClpP], see Fig. 2a). This mutant was designed to reversibly stabilise the tetradecamer through the formation of disulphide bonds between adjacent heptameric rings. The final mutant, which lacks the last 28 residues of CLPP (here termed CLPP^∆C^), was equivalent to the mutant described by Maurizi and colleagues^13^. This mutation creates a more compact and symmetrical heptamer, and hence is proposed to exhibit conventional behaviour on gel filtration^13^. Next, each mutant was generated by site-directed mutagenesis of cDNA encoding mature human CLPP. The different proteins were expressed in *E*. *coli* as Ubiquitin (Ub)-fusion proteins and purified. Following purification of the fusion protein, the H_6_-Ub moiety was cleaved, and the relative purity of the untagged recombinant protein was examined by SDS-PAGE (Fig. S2a). Initially we compared the behaviour of wild type CLPP with each mutant protein using Native-PAGE (Fig. 2b). As described above, wild type mature CLPP formed two distinct species of relatively equal intensity – a heptamer at ~220 kDa and a tetradecamer at ~460 kDa (Fig. 2b, lane 2); both of which were consistent with their theoretical MW (172 kDa and 344 kDa, respectively). In contrast to wild type CLPP, replacement of Arg226 with Ala (which was designed to preclude the formation of a putative salt-bridge across the ring-ring interface) destabilized the tetradecamer significantly, as CLPP^RA^ migrated almost exclusively as the lower species – a heptamer (Fig. 2b, lane 3). Consistent with the importance of this site in the formation of the tetradecamer, the CLPP^ECRC^ mutant migrated solely as a tetradecamer under oxidising conditions (Fig. 2b, lane 4), whilst under reducing conditions (in the presence of TCEP), there was a significant (although incomplete) increase in the amount of heptamer observed (Fig. 2b, lane 5, lower band). Unexpectedly, for reasons that are currently not understood, neither the heptamer nor the tetradecamer of CLPP^∆C^ was resolved by Native-PAGE (Fig. 2b, lane 1). Therefore, we also analysed the mutant proteins using size exclusion chromatography (SEC) (Fig. 2c). Initially, we monitored the elution profile of wild type CLPP (Fig. 2c, top panel). Interestingly, in comparison to the Native-PAGE only a single species was observed for wild type CLPP using SEC. This species however, eluted at ~54 ml, which is equivalent to the elution profile of a ~500 kDa globular protein and is similar to the estimated MW (~460 kDa) of the tetradecamer observed in Native-PAGE. One explanation for the variation in the type of oligomers observed by the two techniques, is that the native electrophoresis conditions promote dissociation of CLPP tetradecamers into heptamers. Nevertheless, we next monitored the elution profile of CLPP^∆C^ (Fig. 2c, second panel). In contrast to our Native-PAGE analysis of CLPP^∆C^, in which we were unable to detect the protein, CLPP^∆C^ migrated as a single symmetrical peak in SEC, with an apparent molecular weight of ~240 kDa (Fig. 2c). The molecular weight of CLPP^∆C^ was not only consistent with published findings^13^ and similar to the theoretical MW of a CLPP heptamer (~172 kDa) but it was also comparable to the observed MW of the heptamer of wild type CLPP in Native-PAGE (Fig. 2b, lower band). Having established the behaviour of wild type CLPP and CLPP^∆C^, we next examined the elution profile of the different CLPP mutants (designed to alter tetradecamer formation). Consistent with our Native-PAGE analysis, CLPP^ECRC^ (under oxidising conditions) eluted from the column in a single symmetrical peak at ~54 ml (equivalent to the tetradecamer), while CLPP^RA^ eluted in a single symmetrical peak at ~61 ml, slightly earlier than the CLPP^∆C^ heptamer peak (~64 ml). This difference in elution volume of the CLPP^RA^ heptamer and the CLPP^∆C^ heptamer is expected to be due to the presence of the exposed C-termini, which increases the hydrodynamic radius of CLPP proposed by Maurizi and colleagues^13^. Consistent with the classification of these peaks, CLPP^ECRC^ (under reducing conditions) eluted in two peaks, one at ~54 ml (equivalent to the tetradecamer) and the other at ~61 ml (equivalent to the heptamer). Collectively, these data demonstrate that reduction of CLPP^ECRC^ (with TCEP) destabilized the tetradecamer, resulting in the formation of the heptamer. The data also confirmed that we can use gel filtration and Native-PAGE to monitor the assembly of CLPP *in vitro*. Significantly, these data suggest that in the absence of CLPX, human CLPP is able to assemble into tetradecamers, under the conditions examined.

### CLPP^Y229D^ displays impaired tetradecamer assembly

**Figure 3:**
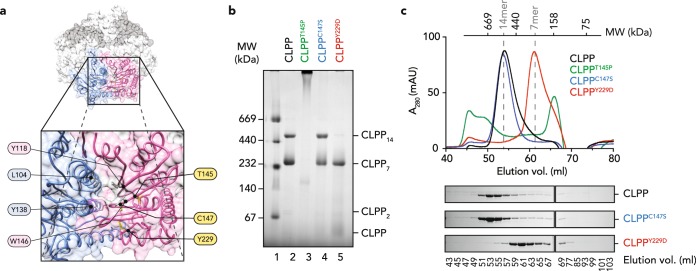
CLPP^Y229D^ displays altered oligomerisation *in vitro*. (**a**) Model showing the surface of Human ClpP_7_ (PDB: 1TG6) highlighting two adjacent subunits (subunit A in blue and subunit B in pink) showing the main chain in “spaghetti”, indicating the position of the Hp residues L104 (blue) and Y138 (blue) on subunit A and Y118 (pink) and W146 (pink) on subunit B. The relative position of the residues that are mutated in Perrault syndrome (T145, C147 and Y229) and analysed in this study are indicated in yellow. (**b**) Assembly of wild type CLPP and CLPP Perrault mutants under native conditions. Recombinant, untagged wild type or mutant CLPP (4 µg) was separated by Native-PAGE and visualised by staining with CBB. The oligomeric composition of wild type human CLPP (lane 2) was compared to CLPP^T145P^ (lane 3), CLPP^C147S^ (lane 4) and CLPP^Y229D^ (lane 5). The tetradecamer (CLPP_14_) and heptamer (CLPP_7_) are indicated, as is the monomer (CLPP) and dimer (CLPP_2_) of CLPP^Y229D^. (**c**) The high order oligomeric complexes of recombinant, untagged wild type or mutant CLPP were separated by size exclusion chromatography (SEC) using a Superdex 200 HiLoad 16/60 pg column (GE Healthcare). Elution profiles of wild type CLPP (black line and top panel), CLPP^T145P^ (green line), CLPP^C147S^ (blue line and middle panel) and CLPP^Y229D^ (red line and bottom panel) were measured at 280 nm (A_280_). Proteins from the indicated fractions were separated by SDS-PAGE and visualised by staining with CBB. Lines indicate the peak elution volume of thyroglobulin (669 kDa), ferritin (440 kDa), aldolase (158 kDa) and conalbumin (75 kDa). The full-length gels for SEC are presented in Supplementary Fig. S8.

Next, having established that we could use size exclusion chromatography and Native-PAGE to monitor the oligomeric state of mature human CLPP *in vitro*, we examined the oligomeric state of the different Perrault mutant proteins (Fig. 3a, highlighted in yellow). To do so, we first introduced the appropriate mutation into cDNA coding for mature human CLPP, then overexpressed each protein in *E*. *coli*. Following affinity isolation, cleavage and recovery of the untagged proteins, we examined the relative purity of each mutant by SDS-PAGE (Fig. S2b). Although two of the mutant proteins (CLPP^C147S^ and CLPP^Y229D^) could be purified to homogeneity (>95% purity), the third mutant (CLPP^T145P^) could only be recovered to ~85% purity (Fig. S2b, lane 3). While expression of CLPP^T145P^ in ∆*dnaK* (EN2) *E*. *coli* cells was successful in removing one impurity – DnaK, despite numerous attempts using a variety of different approaches, two impurities remained. These impurities are anticipated (based on their apparent molecular weights of ~60 and 40 kDa, respectively) to be GroEL and DnaJ and hence human CLPP^T145P^ likely has a folding defect.

Initially, to test the structural integrity of each CLPP mutant, we monitored the ability of wild type CLPP to form an oligomer and compared its assembly to the different mutant proteins. To do so, we performed non-specific chemical crosslinking using glutaraldehyde (GA) as described previously^13^. As expected, wild type CLPP formed a ladder of oligomeric species in the presence of GA (Fig. S3, lanes 2–4). Consistent with wild type CLPP, both CLPP^C147S^ (Fig. S3, lanes 8–10) and CLPP^Y229D^ (Fig. S3, lanes 11–13) formed a ladder of seven discrete crosslinked protein bands. In contrast, these discrete oligomeric species were not observed with CLPP^T145P^ (Fig. S3, lanes 5–7). Collectively, these data suggest that in the absence of CLPX, CLPP^T145P^ is unable to assemble into a heptamer. Next, we examined the ability of these proteins (in the absence of crosslinker) to form heptamers and higher order complexes. Consistent with our previous findings (Fig. 2), wild type CLPP migrated as two discrete bands in Native-PAGE, a heptamer at ~220 kDa and a tetradecamer at ~460 kDa (Fig. 3b, lane 2). Similar to wild type CLPP, both bands were observed for CLPP^C147S^ (Fig. 3b, lane 4). In contrast, only the lower MW species (the heptamer) was observed for CLPP^Y229D^ (Fig. 3b, lane 5). This suggests that the ring-ring interface of CLPP^Y229D^ was compromised and given that a small amount of CLPP^Y229D^ monomers and dimers were also observed in the gel (Fig. 3b, lane 5), it appears that this mutation (in contrast to the other mutants tested) may also affect intra-ring stability. Notably, in contrast to CLPP^C147S^ and CLPP^Y229D^, the majority of CLPP^T145P^ failed to enter the separating gel, suggesting that CLPP^T145P^ has a propensity to aggregate *in vitro* (Fig. 3b, lane 3). Finally, we examined that ability of each mutant to oligomerise in solution, using gel filtration (Fig. 3c). Consistent with Native-PAGE analysis of the different mutants, CLPP^C147S^ (Fig. 3c, blue) eluted in a symmetrical peak at ~53 ml (equivalent to the elution volume of a CLPP tetradecamer; Fig. 3c, black), while CLPP^Y229D^ (Fig. 3c, red) eluted in a peak at ~61 ml (equivalent to the elution volume of the CLPP heptamer). In contrast, CLPP^T145P^ (Fig. 3c, green) eluted in multiple peaks, with much of the protein eluting near the void volume of the column or as a monomer. Collectively, these data demonstrate that CLPP^Y229D^ exhibits a mild defect in CLPP assembly, while mutations in the Hp have quite different effects on protein assembly (Fig. 3). Interestingly, although one Hp mutant (CLPP^T145P^) was unable to assemble into its native oligomer (likely due to a folding defect), the assembly of the other Hp mutant (CLPP^C147S^) was unaffected (Fig. 3). As such, PRLTS3 mutations, even different mutations within the same region, are likely to have broadly different effects on proteolytic activity.

### PRLTS3 CLPP mutants demonstrate varied peptidase activities

**Figure 4:**
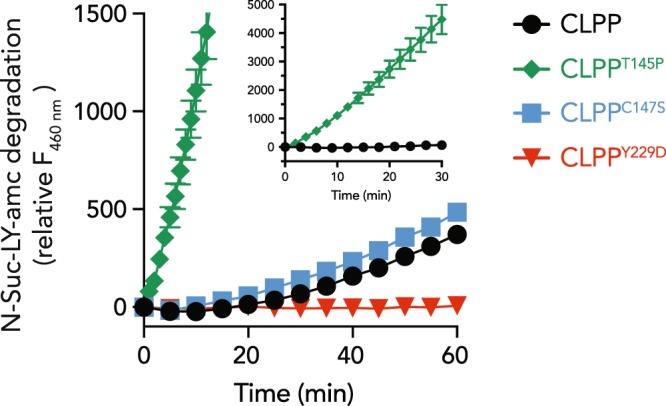
CLPP^T145P^ displays enhanced peptidase activity. The turnover of the fluorogenic peptide substrate Suc-LY-amc (1 mM) was monitored in the presence of either wild type CLPP (black circles), CLPP^T145P^ (green diamonds), CLPP^C147S^ (blue squares) or CLPP^Y229D^ (red triangles). The cleavage of Suc-LY-amc was monitored by fluorescence (excitation = 380 nm, emission = 460 nm). Error bars represent the standard error of the mean (SEM) of three independent experiments.

Next, we examined the peptidase activity of the different PRLTS3 mutant proteins. Initially we screened the peptidase activity of wild type CLPP, in the absence of its cognate unfoldase CLPX, using fluorescently labelled peptides. Consistent with the recent findings of Sieber and colleagues^41^, we discovered that human CLPP displayed weak but measurable peptidase activity using the fluorescently labelled peptide Suc-LY-amc (Fig. 4, black circles). Therefore, we tested the peptidase activity of each CLPP mutant using this substrate. As predicted, due to proximity of the mutation to the active site, the peptidase activity of CLPP^Y229D^ was completely abolished (Fig. 4, red triangles). Importantly, this loss of activity, by CLPP^Y229D^, validates Suc-LY-amc as a CLPP substrate. In contrast to CLPP^Y229D^, both Hp mutants (CLPP^T145P^ and CLPP^C147S^) displayed peptidase activity towards the peptide substrate, even though they each showed substantial differences in assembly (Fig. 3). One mutant, CLPP^C147S^, exhibited a similar peptidase activity to wild type CLPP (Fig. 4, blue squares), while the peptidase activity of the other Hp mutant (CLPP^T145P^) was stimulated by ~20-fold (Fig. 4, green diamonds). These data demonstrate that PRLTS3 mutations in CLPP exhibit broadly different effects on peptidase activity, from complete loss of function to enhanced activity. Although the “hyper-activation” of CLPP^T145P^ was quite unexpected, it does share some similarities with a “gain-of-function” mutant recently described for *Sa*ClpP, in which Y63 (in the Hp) was replaced with alanine^42^. Interestingly, based on the structure of *Sa*ClpP, Y63 (found in β strand 3) is located only ~3.5 Å from the highly conserved residue T90 (located in the adjacent strand, β strand 5), which is equivalent to T145 in human CLPP. Hence, we propose that structural rearrangement of β strand 5 (caused by the T145P mutation) may affect the adjacent strand (β strand 3) in a similar fashion to that observed for the *Sa*ClpP^Y63A^ mutation^42^. Collectively, our data suggest that mutations within, or around, the Hp of CLPP can have distinct effects on peptide substrate recognition and/or cleavage. Therefore, different point mutations within this region are likely to exhibit different phenotypes. Indeed, based on our data, in addition to the predicted dysregulation of CLPX-mediated degradation by CLPP, the T145P mutation in CLPP may result in a toxic gain-of-function in mammalian cells. Consistent with this idea, patients with the T145P mutation exhibit profound deafness and severe neurological symptoms^4,43^. As such, analysis of the different pathogenic mutations in CLPP might provide evidence for a link to the severity of the disease observed in different Perrault syndrome patients.

### CLPP^Y229D^ and CLPP^T145P^ exhibit decreased interaction with human CLPX

**Figure 5:**
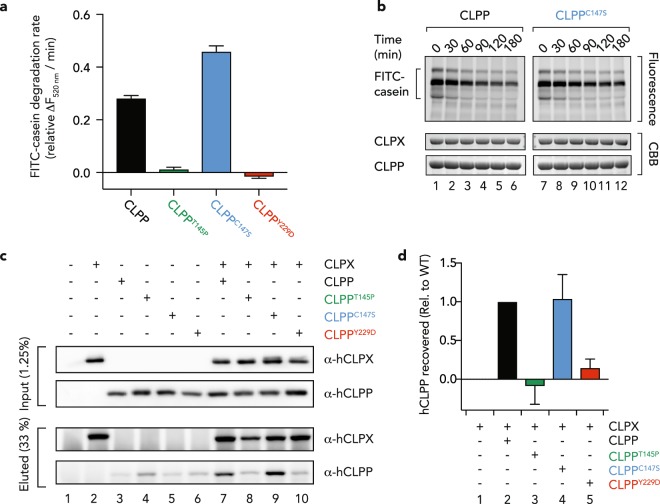
CLPP^T145P^ and CLPP^Y229D^ abolish functional association with human CLPX. (**a**) Human CLPX-dependent turnover of FITC-casein was monitored by a change in fluorescence at 520 nm [excitation = 490 nm] in the presence of either wild type CLPP (black bar), CLPP^T145P^ (green bar), CLPP^C147S^ (blue bar) or CLPP^Y229D^ (red bar). The change in fluorescence at 520 nm (ΔF_520 nm_) of FITC-casein was calculated relative to the initial fluorescence of the substrate from three independent experiments. Error bars represent SEM. (**b**) To monitor the turnover of FITC-casein directly, samples as described in (**a**) containing either wild type CLPP (lanes 1–6) or CLPP^C147S^ (lanes 7–12) were separated by SDS-PAGE and monitored by fluorescence (upper panel). As a loading control, the levels of CLPX and CLPP (in each reaction) were monitored by staining with CBB (lower panels). (**c**) To monitor the interaction between human CLPX and CLPP, human CLPX was immunoprecipitated (IP) using a specific anti-CLPX antisera, in the absence (lanes 2) or presence (lanes 7–10) of wild type or mutant human CLPP. To ensure the specificity of the co-immunoprecipitation (co-IP) the recovery of wild type and mutant human CLPP was also monitored in the absence of human CLPX (lanes 3–6). Following co-IP of human CLPP, the input (1.25%) and the eluted (33%) proteins were separated by SDS-PAGE and transferred to PVDF, before being immunodecorated with specific antisera, as indicated. (**d**) Quantitation of human CLPP recovered in CLPX coIP (as described in (**c**) in which non-specific interaction (lanes 3–6, respectively) was subtracted from the specific interaction (lanes 7–10, respectively)) of three independent experiments. Error bars represent SEM. The full-length gels for (**b**) are presented in Supplementary Fig. S9 and the full-length western blot for (**c**) are presented in Supplementary Fig. S10.

Finally, we examined the ability of each mutant protein to interact with its cognate unfoldase (Fig. 5). Initially this was performed using an indirect assay, in which we examined the turnover of a CLPX-dependent substrate, the model unfolded protein casein. In this instance we used fluorescently labelled casein (FITC-casein) and monitored the change in FITC fluorescence as a measure of the rate of CLPXP-mediated turnover (Fig. 5a). As expected, the CLPXP-mediated turnover of FITC-casein (Fig. 5a, black bar and Fig. 5b, lanes 1–6) was dependent on the addition of ATP (Fig. S4). Consistent with peptidase activity of CLPP^C147S^ (Fig. 4a), the C147S mutant also facilitated the CLPX-mediated degradation of FITC-casein at a similar rate to that of wild type CLPP (Fig. 5a, blue bar and Fig. 5b, lanes 7–12). In contrast to wild type CLPP and CLPP^C147S^, neither CLPP^T145P^ (Fig. 5a, green bar) nor CLPP^Y229D^ (Fig. 5a, red bar) were able to facilitate the turnover of the model unfolded protein. In the case of CLPP^T145P^ (which exhibits increased peptidase activity), this loss of CLPX-mediated turnover appears to be due to a compromised interaction with the unfoldase component. However, in the case of CLPP^Y229D^, it remained unclear if the CLPP mutant had lost or retained the ability to interact with CLPX. To determine which mutant retained the ability to interact with human CLPX, we performed a series of immunoprecipitation experiments. In this case, we immobilised CLPX antisera to Protein A Sepharose (PAS) and then incubated the immobilised antibody with wild type or mutant CLPP in the absence or presence of CLPX. Following incubation of the recombinant proteins with the beads and removal of any non-specifically bound proteins from the PAS through extensive washing, the interacting proteins were eluted, and the samples separated by SDS-PAGE before being transferred to PVDF membrane for analysis via immunoblotting with specific antisera (Fig. 5c). Despite some weak non-specific binding of wild type CLPP to the beads (Fig. 5c, lane 3 lower panel), there was a significant increase in the recovery of CLPP in the presence of CLPX (Fig. 5c, lane 7), demonstrating a specific interaction between CLPX and CLPP. Consistent with the interaction observed for wild type CLPP, CLPP^C147S^ also exhibited some weak non-specific binding to the beads (Fig. 5c, lane 5), with a significant increase in its recovery in the presence of CLPX (Fig. 5c, lane 9). In contrast to CLPP^C147S^, there was little recovery of either CLPP^T145P^ (Fig. 5c, lane 8) or CLPP^Y229D^ (Fig. 5c, lane 10) in the presence of CLPX. To determine the relative level of specific interaction between CLPX and each of the mutants, we quantitated the recovery of CLPP (wild type and mutant) in the presence and absence of CLPX, subtracting the amount of non-specific binding (i.e. in the absence of CLPX) (Fig. 5d). These data demonstrate that CLPP^C147S^ binds to CLPX with a similar affinity to wild type CLPP, while in contrast neither CLPP^T145P^ (Fig. 5d, green bar) nor CLPP^Y229D^ (Fig. 5d, red bar) showed any significant interaction with CLPX. Taken together, the data demonstrate that the lack of CLPX-mediated degradation of FITC-casein by CLPP^T145P^ is due to a loss of interaction with CLPX, while in contrast the lack of CLPX-mediated degradation of FITC-casein by CLPP^Y229D^ is likely due to a combined effect of a loss of peptidase activity as well as a loss of interaction with CLPX. Interestingly, despite the loss of CLPP^T145P^ interaction with human CLPX, we also the examined (indirectly) the ability of CLPP^T145P^ to interact with ecClpX. This was performed by monitoring the ecClpX-mediated turnover the model “folded” substrate (GFP-ssrA). Incredibly, CLPP^T145P^ retained the ability to facilitate the ecClpX-mediated the turnover of GFP-ssrA (Fig. S5, green diamonds) at a similar rate to both wild type CLPP (Fig. S5, black circles) and CLPP^C147S^ (Fig. S5, blue squares). In contrast, CLPP^Y229D^ (which lacks peptidase activity) was unable to facilitate the ecClpX-mediated turnover of GFP-ssrA (Fig. S5, red circles). Consistent with these data, Sieber and colleagues^41^ recently showed that CLPP^T145P^ was able to facilitate the ecClpX-dependent turnover of GFP-ssrA at a similar rate to wild type CLPP. Despite our validation of this activity (for CLPP^T145P^), the molecular mechanism of GFP-ssrA turnover (mediated by ecClpX) remains unclear. Significantly, these data demonstrate that although CLPP^T145P^ can interact with ecClpX, it is unable to interact with human CLPX. This unexpected result reinforces the importance of examining homogeneous systems to study the effect of pathogenic mutations.

## Conclusion

Although the physiological role of mitochondrial CLPP (and CLPX) remains poorly understood, a handful of studies suggest that mammalian CLPP similar to its bacterial homologs, plays a crucial role^10,44–46^. Consistently, a number of recent studies have linked mutations in human CLPP with PRLTS3^2,4,19–21^. Importantly, PRLTS3 patients share a number of phenotypes (such as deafness and infertility in both sexes) with *CLPP* null mice (*CLPP*^−/−^) which provides strong support for a link between the mutations in *CLPP* and PRLTS3. Interestingly, in addition to the shared phenotypes (between PRLTS3 patients and *CLPP*^−/−^ mice), PRLTS3 patients also exhibit some major neurological impairments such as epilepsy, microcephaly or learning difficulties, which suggests that some of the pathogenic mutations in CLPP might trigger a toxic gain-of-function in the mitochondrion.

In this study, we have dissected the biogenesis (import, processing and assembly) and activity (with and without its cognate unfoldase, CLPX) of three different PRLTS3 associated mutants of CLPP *in vitro*. Although two mutants (CLPP^T145P^ and CLPP^Y229D^) displayed significant defects in CLPP assembly and/or activity, one mutant (CLPP^C147S^) quite unexpectedly failed to display a single defect in any of the activities examined. Significantly, although this mutation is located within the Hp of CLPP (required for interaction with CLPX), a defect in CLPX-interaction or CLPX-mediated degradation of either a folded or unfolded substrate was not observed. One possible explanation for this surprising result is that CLPP^C147S^ may exhibit a specific defect in the recognition and/or translocation (into CLPX) of a unique substrate such as ERAL1 that was recently linked to the PRLTS^9,47^, or a currently unknown substrate that may be associated with PRLTS. An alternate explanation is that the mutant protein is unstable *in vivo*, and hence the levels of CLPP drop below a minimum threshold concentration required for normal cellular function. Consistent with this idea, a splice mutation in *CLPP* (which reduces CLPP expression) is known to cause PRLTS3^4^. Significantly, the C147S mutation was previously observed to display a decreased melting temperature^41^, hence in contrast to other Hp mutations, this mutation may affect the physiological levels of CLPP. Collectively, these findings are consistent with relatively mild symptoms observed in patients with the C147S mutation, although the extent of gonadal dysgenesis was not completely examined in these patients^4^. The final possibility is that PRLTS3 patients (with the C147S mutation) is caused not by the mutation in CLPP, but rather by an unidentified mutation within a novel gene. In support of this idea, C147 (in contrast to T145 and Y229) is only moderately conserved across ClpP sequences (see Fig. S6). Indeed, at this position (i.e. the equivalent of position 147 in CLPP) the pathogenic residue – Ser – is permissive in several ClpP homologs, including *P*. *falciparum* ClpP (see Fig. S6). Therefore, in order to better understand the molecular basis of PRLTS3 caused by CLPP^C147S^, the physiological levels (and/or half-life) of CLPP in these patients will need to be examined.

In contrast to C147S, the two remaining mutations (T145P and Y229D) are located at highly conserved positions within the protein – T145 is almost completely conserved amongst ClpP sequences, while Y229 is restricted to large hydrophobic residues (see Fig. S6). Consistent with the highly conserved nature of these residues, PRLTS3 patients with mutations in these residues display acute developmental defects (e.g. microcephaly, learning difficulties and muscle spasticity) in addition to SNHL and infertility. Not surprisingly, both proteins (CLPP^T145P^ and CLPP^Y229D^) exhibit severe defects in a range of biochemical activities. In addition to the loss of all human CLPX-mediated activities by these mutants, the peptidase activity of CLPP^Y229D^ was also abolished, while in contrast the peptidase activity of CLPP^T145P^ was enhanced, at least for some substrates (Fig. 4). Interestingly, although we observed an enhanced activity for the “poor” substrate (Suc-LY-amc), a slight reduction in the peptidase activity of CLPP^T145P^ was reported by Sieber and colleagues^41^ for an optimised substrate (Ac-Phe(3,4-Cl_2_)-hArg-Leu-ACC). In contrast, neither group observed a change in the proteolytic activity of CLPP^T145P^ (in the presence of ecClpX) using the substrate GFP-ssrA (Fig. S5)^41^. Collectively, these data demonstrate the importance of studying homogeneous systems when studying the effect of a pathogenic mutation. Moreover, they also appear to suggest that CLPP^T145P^ exhibits an altered substrate specificity. Nevertheless, in addition to the altered specificity of CLPP^T145P^, both mutants (CLPP^T145P^ and CLPP^Y229D^) display defects in folding and/or assembly. Hence, the additional phenotypes observed in patients carrying these mutations might be caused by the accumulation of incompletely folded or unassembled mutant proteins. This could be caused either directly, through the accumulation of misfolded or aggregated protein in the mitochondrion or indirectly through the sequestration of important molecular chaperones. Indeed, protein misfolding and aggregation is an underlying cause of several neurodegenerative diseases^48,49^. Likewise, reduced chaperone activity of HSP60 has been associated with an autosomal dominant form of spastic paraplegia (SPG13)^50^. Therefore, we propose that PRLTS3 patients carrying mutations in CLPP that only affect the cellular levels of CLPP (and not CLPP function) are likely to manifest in mild forms of the disease. In contrast, mutations in CLPP (such as the T145P and Y229D), that affect the folding and/or assembly of CLPP, may exhibit a toxic gain-of-function as a result of the reduced proteostatic capacity of the organelle. While the introduction of different pathogenic mutations into *CLPP*^−/−^ mice should help to determine if gain-of-function mutations contribute to the pathogenesis of the disease, the identification of validated human CLPP substrates could be very helpful in identifying a critical loss-of-function. Significantly, the recent identification of ERAL1 (an assembly chaperone for the mitochondrial ribosome associated with PRLT6) as a putative CLPP substrate suggests that PRLTS3 arises from a defect in mitochondrial protein translation. Consistent with this idea, several other forms of PRLTS have also been associated with mutations in genes involved in mitochondrial translation. Hence, in the future it will be interesting to examine if the PRLTS3 associated CLPP mutants affect ribosome assembly in the mitochondrion.

## Electronic supplementary material

Supplementary Information
