# Supplementary material for: Perrault syndrome type 3 caused by diverse molecular defects in CLPP
Source: Sci Rep. 2018 Aug 27;8:12862. doi: 10.1038/s41598-018-30311-1 (PMC6110781; doi:10.1038/s41598-018-30311-1)
Supplement: Supplementary file 1 — Supplementary Information [file 41598_2018_30311_MOESM1_ESM.pdf]

Perrault syndrome type 3 caused by diverse molecular defects in CLPP.

Erica J. Brodie[1,§], Hanmiao Zhan[1], Tamanna Saiyed[1], Kaye N. Truscott[1] and David A. Dougan[1]

[1]Department of Biochemistry and Genetics, La Trobe Institute for Molecular Science, La Trobe University, Melbourne, 3086, Victoria, Australia.

[§]Present address: Department of Immunology and Pathology, Monash University, Melbourne, 3004, Victoria, Australia

Correspondence and requests for materials should be addressed to D.A.D. (email: d.dougan@latrobe.edu.au) or K.N.T. (email: k.truscott@latrobe.edu.au)

Keywords: Perrault syndrome, PRLTS3, CLPP, mitochondrial proteostasis.

Materials and Methods

Glutaraldehyde Crosslinking

Prior to crosslinking, recombinant protein samples were dialysed for at least 2 h at 4 °C, in dialysis buffer (50 mM MOPS-KOH [pH 7.5], 300 mM NaCl, 0.025% (v/v) Triton X-100, 10% (v/v) glycerol, in the presence or absence of 1 mM DTT, as required). Crosslinking of CLPP (0.16 µg/µl) was performed at 25 °C, in Crosslinking (XL) buffer (50 mM HEPES-KOH [pH 7.5], 300 mM NaCl, 200 mM KCl, 20 mM $MgCl_2$, 0.025% (v/v) Triton X-100, 10% (v/v) glycerol, in the presence or absence of 1 mM DTT, as required). The reaction was initiated with the addition of 0.1% (v/v) glutaraldehyde, aliquots were taken at the indicated time points and the reaction stopped with the addition of saturated Tris, followed by the addition of sample buffer before the sample was heat-treated (95 °C, 5 min).

Table S1: Primer sequences used to introduce point mutations into pHUE/*CLPP* and *pOTB7/CLPP* via site-directed mutagenesis.

**Primer**	**Gene**	**DNA sequence (5′ – 3′)**	**Primer features**
T145P_1 (fwd)	*CLPP (T145P)*	GGCAGATCGGGTTGAGGATGTACTGCATCG	Introduces ACC>CCC (p.T145P) point mutation into human *CLPP* using Phusion site-directed mutagenesis, includes an *Nco* I restriction enzyme site for screening
T145P_2 (rev)	*CLPP (T145P)*	CATGGTGCGTGGCCAGGCCGCCAGCATG	
C147S_1 (fwd)	*CLPP (C147S)*	GTGCATATGGGGTTGAGGATGTACTGCATC	Introduces TGC>AGT (p.C147S) point mutation into human *CLPP* using Phusion site-directed mutagenesis, includes a *Nde* I restriction enzyme site for screening
C147S_2 (rev)	*CLPP (C147S)*	CTGGAGTGTGGGCCAGGCCGCCAGCATGGGC	
Y229D_Fwd	*CLPP (Y229D)*	CGCGACATGAGCCCCATGGAGGCCCAGGAG	Introduces TAC>GAC (p.Y229D) point mutation into human *CLPP* using Phusion site-directed mutagenesis, includes a *Pvu* I restriction enzyme site for screening
Y229D_Rev	*CLPP (Y229D)*	ATCGCGCTCCATGGCGGACTCGATCACCTG	
hP_ΔC31	*CLPPΔC*	CCCCAGGACGGTGAGGATGAGCCCAC	Introduces TAA point mutation into human *CLPP* (which encodes deletion of last 30 residues from the C-terminus of CLPP) using Phusion site-directed mutagenesis
hP_ΔC31 rev	*CLPPΔC*	TTAGTGGACCAGAACCTTGTCTAAGATG	
hP RA fwd	*CLPP (R226A)*	GCCGATCGCTACATGAGCCCCATGGAGGC	Introduces AGG>GCC (p.R226A) point mutation into human *CLPP* using Phusion site-directed mutagenesis, includes a *Pvu* I restriction enzyme site for screening
hP WT rev	*CLPP (R226A)*	CTCCATGGCGGACTCGATCACCTGCAGGCTC	
hP RC fwd	*CLPP (R226C)*	TGCGATCGCTACATGAGCCCCATGGAGGC	Introduces GAG>TGC (p.E225C) and AGG>TGC (p.R226C) point mutations into human *CLPP* using Phusion site-directed mutagenesis, includes a *Pvu* I restriction enzyme site for screening
hP EC rev	*CLPP (E225C)*	GCACATGGCGGACTCGATCACCTGCAGGCTC	

Table S2: plasmids used in this study.

Plasmid name	DNA (insert)	Plasmid features, reference
pDT2772	*Human m-CLPP*	pHUE [Bezawork-Gelata et al., 2015]
pDT3001	*Human m-CLPP*	pDT2772/*CLPP(C147S)*
pDT3002	*Human m-CLPP*	pDT2772/*CLPP(T145P)*
pDT3384	*Human m-CLPP*	pDT2772/*CLPP(Y229D)*
pDT3033	*Human m-CLPP∆C*	pDT2772/*CLPP∆C*
pDT3527	*Human m-CLPP*	pDT2772/*CLPP(R126A)*
pDT3529	*Human m-CLPP*	pDT2772/*CLPP(E125C/R126C)*
pDT2015	*Human CLPP*	pOTB7 (IMAGE clone 3542292)
pDT3268	*Human CLPP*	pDT2015/*CLPP(C147S)*
pDT3269	*Human CLPP*	pDT2015/*CLPP(T145P)*
pDT3391	*Human CLPP*	pDT2015/*CLPP(Y229D)*
pDD173	*GFP-ssrA*	pUHIS [Dougan et al., 2002]
pDD250	*E. coli clpX (ec clpX)*	pNHIS [Dougan et al., 2003]

References

Bezawork-Geleta, A., Saiyed, T., Dougan, D. A. & Truscott, K. N. Mitochondrial matrix proteostasis is linked to hereditary paraganglioma: LON-mediated turnover of the human flavinylation factor SDH5 is regulated by its interaction with SDHA. *FASEB J* **28**, 1794-1804, doi:10.1096/fj.13-242420 (2014).

Dougan, D. A., Reid, B. G., Horwich, A. L. & Bukau, B. ClpS, a substrate modulator of the ClpAP machine. *Mol Cell* **9**, 673-683 (2002).

Dougan, D. A., Weber-Ban, E. & Bukau, B. Targeted delivery of an ssrA-tagged substrate by the adaptor protein SspB to its cognate AAA+ protein ClpX. *Mol Cell* **12**, 373-380 (2003).

Supplementary Figures.

Figure S1. Radiolabelled wild type CLPP precursor (lane 2 – 4), was imported into mitochondria isolated from HeLa cells, in the presence or absence of a membrane potential (Δψ) as indicated. The precursor (pre) protein was processed into an intermediate (i-) and

finally mature (m-) CLPP. The radiolabelled proteins were separated by 12.5% SDS-PAGE and visualised by (**a**) autoradiography alongside recombinant m-CLPP ($CLPP_{53\text{-}277}$; lane 5) which was visualised by (**b**) staining with CBB. **c.** To compare the molecular weight of the purified recombinant CLPP with the radiolabelled imported m-CLPP the autoradiograph was overlaid onto the CBB image.

Figure S2. a. Relative purity of wild type CLPP (lane 1) with $CLPP^{RA}$ (lane 2), $CLPP^{ECRC}$ (lane 3) or $CLPP^{\Delta C}$ (lane 4). **b.** Comparison of the relative purity of wild type CLPP (lane 2) with $CLPP^{T145P}$ (lane 3), $CLPP^{C147S}$ (lane 4) or $CLPP^{Y229D}$ (lane 5). Proteins were separated via 16.5 % Tricine-buffered SDS-PAGE and visualised by staining with CBB.

Figure S3. The oligomeric assembly of recombinant CLPPs (50 µg) was analysed by PAGE following glutaraldehyde (GA) crosslinking. Following incubation (at 25 °C) of wild type CLPP (lanes 2 – 4), $CLPP^{T145P}$ (lanes 5 – 7), $CLPP^{C147S}$ (lanes 8 – 10) or $CLPP^{Y229D}$ (lanes 11 – 13) with 0.1 % GA, the proteins were separated by 5 – 12.5 % MES-buffered SDS-PAGE and visualised by staining with CBB.

Figure S4. The hCLPXP-mediated turnover of FITC-casein was monitored in the presence (lanes 1 – 6) or absence (lanes 7 – 12) of ATP, for the indicated times. To visualise FITC-casein, the proteins were separated by SDS-PAGE and FITC-casein was visualised by fluorescence (Excitation 490 nm, Emission 520 nm).

Figure S5. The ecClpX-mediated turnover of GFP-ssrA was monitored by fluorescence at 510 nm (excitation at 400 nm) in the absence of additional components (white squares) or in the presence of either wild type ecCLPP (black squares), wild type hCLPP (black circles), $hCLPP^{T145P}$ (green diamonds), $CLPP^{C147S}$ (blue squares) or $CLPP^{Y229D}$ (red circles). Error bars represent SEM (n=3).

Figure S6. Partial protein sequences of *Homo sapiens* ClpP (Q16740), *Mus musculus* ClpP (O88696), *Plasmodium falciparum* ClpP (O97252), *Escherichia coli* ClpP (P0A6G7), *Caulobacter crescentus* ClpP (P0CAU1), *Agrobacterium tumefaciens* (*Atu*) ClpP1 (Q8UEX6), *Atu* ClpP2 (Q8UFY6), *Mycobacterium tuberculosis* (*Mtb*) ClpP1 (P9WPC5), *Mtb* ClpP2 (P9WPC3), *Corynebacterium glutamicum (Cgl)* ClpP1 (Q8NN02), *Cgl* ClpP2 (Q8NN01), *Arabidopsis*

thaliana (*Ath*) ClpP1 (P56772), *Ath* ClpP2 (Q9FN42), *Ath* ClpP3 (Q9SXJ6), *Ath* ClpP4 (Q94B60), *Ath* ClpP5 (Q9S834), *Zea mays* (P26567), *Salmonella typhimurium* ClpP (P0A1D7), *Yersinia pestis* ClpP (Q66DT4), *Vibrio cholera* ClpP (Q9KQS6), *Pseudomonas aeruginosa* (*Psa*) ClpP1 (Q9I2U1), *Psa* ClpP2 (Q9HYR9), *Haemophilus influenzae* ClpP (P43867), *Thermotoga maritima* ClpP (Q9WZF9), *Bacillus subtilis* ClpP (P80244), *Staphylococcus aureus* ClpP (P63786), *Lactococcus lactis* ClpP (Q9ZAB0), *Lactobacillus acidophilus* ClpP (Q5FL55) were obtained from Uniprot and aligned with ClustalX. Numbering refers to the human CLPP sequence. Red numbers indicated residues that have been mutated in patients with PRLTS3. (*) - indicates identical residues; (**:**) - indicates highly conserved residues; (**.**) - indicates conserved residues. The bar (below the alignment) represents the relative conservation of each residue.

Figure S7. Full-length gels of Figure 1b showing radiolabelled precursor protein of $CLPP^{T145P}$ (lanes 1 – 5), $CLPP^{C147S}$ (lanes 6 – 10), $CLPP^{Y229D}$ (lanes 11 – 15) and wild type CLPP (lane 16 – 20), was imported into mitochondria isolated from HeLa cells, in the presence or absence of a membrane potential (Δψ) as indicated. The precursor (pre) protein was processed into an intermediate (i-) and finally mature (m-) CLPP. All radiolabelled proteins were separated by 12.5% SDS-PAGE and visualised by digital autoradiography.

Figure S8. Full-length gels of Figure 3c showing wild type or mutant CLPP, separated by size exclusion chromatography (SEC) using a Superdex 200 HiLoad 16/60 pg column (GE Healthcare). Proteins were separated by SDS-PAGE and visualised by staining with CBB.

Figure S9. Full-length gels of Figure 5b showing the CLPX-mediated turnover of FITC-casein by wild type CLPP (lanes 1 – 6) or $CLPP^{C147S}$ (lanes 8 – 13), monitored by fluorescence (upper panel) following separation by SDS-PAGE. As a loading control, the levels of CLPX and CLPP (in each reaction) were monitored by staining with CBB (lower panel).

Figure S10. Full-length blots of Figure 5c examining the interaction between human CLPX and CLPP. Human CLPX was immunoprecipitated (IP) using a specific anti-CLPX antisera, in the absence (lanes 2) or presence (lanes 7 – 10) of wild type or mutant human CLPP. To ensure the specificity of the co-immunoprecipitation (co-IP) the recovery of wild type and mutant human CLPP was also monitored in the absence of human CLPX (lanes 3 – 6). Following co-IP of human CLPP, the input (1.25 %) and the eluted (33 %) proteins were separated by SDS-

PAGE and transferred to PVDF, before being immunodecorated first with anti-CLPP (left panel) and then with anti-CLPX (right panel).

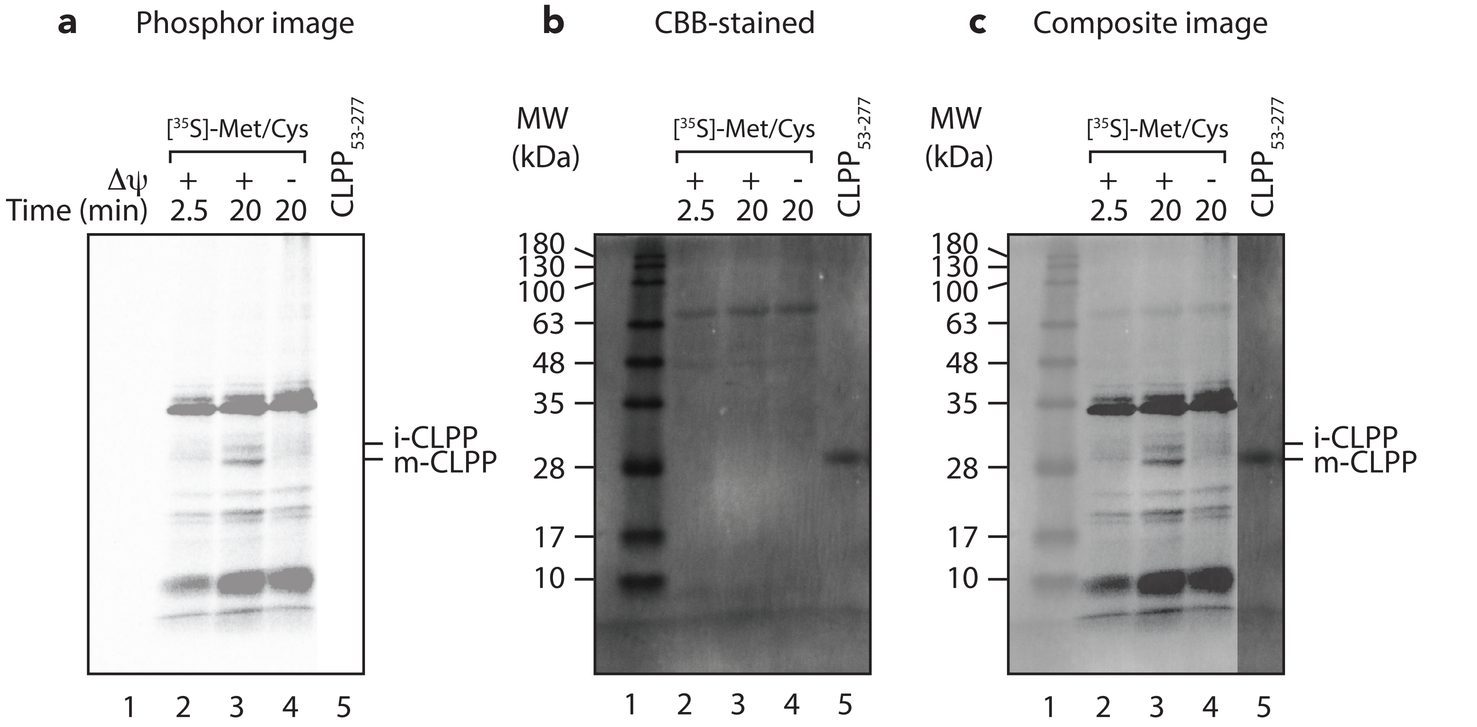

Figure S1 Brodie et al.,

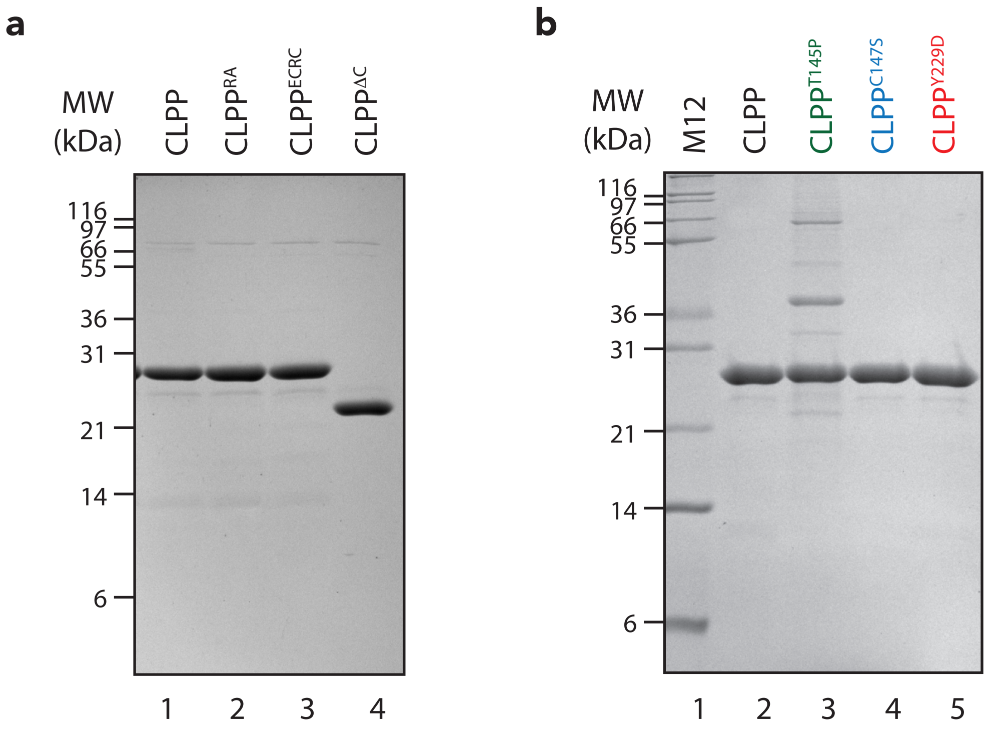

Figure S2 Brodie et al.,

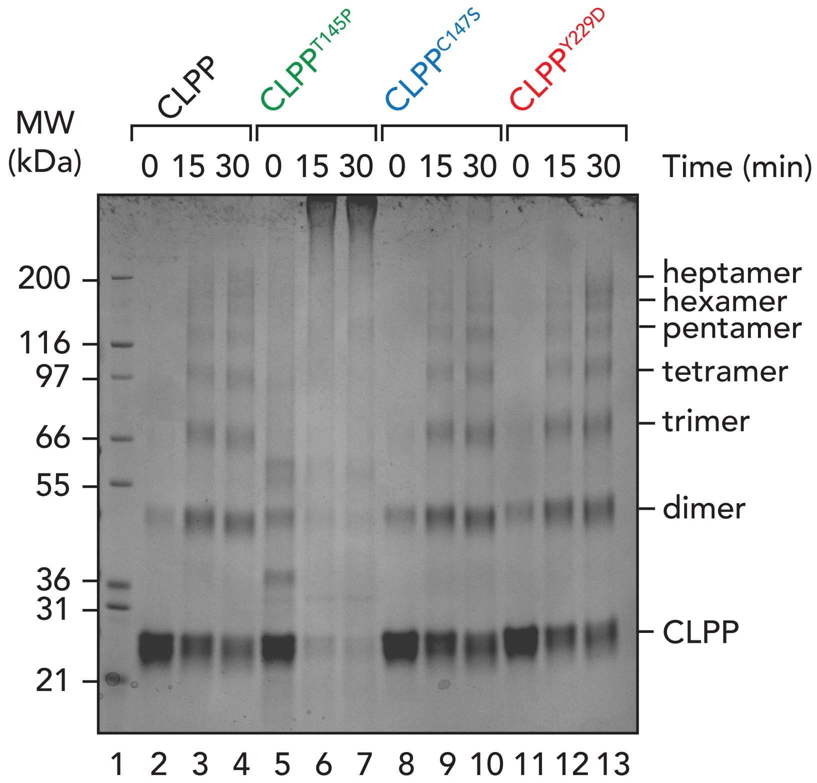

Figure S3 Brodie et al.,

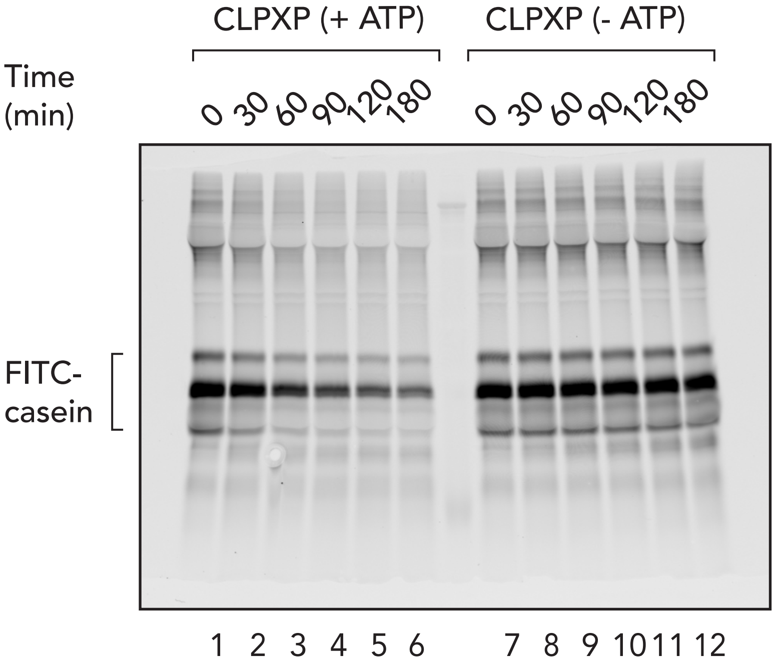

Figure S4 Brodie et al.,

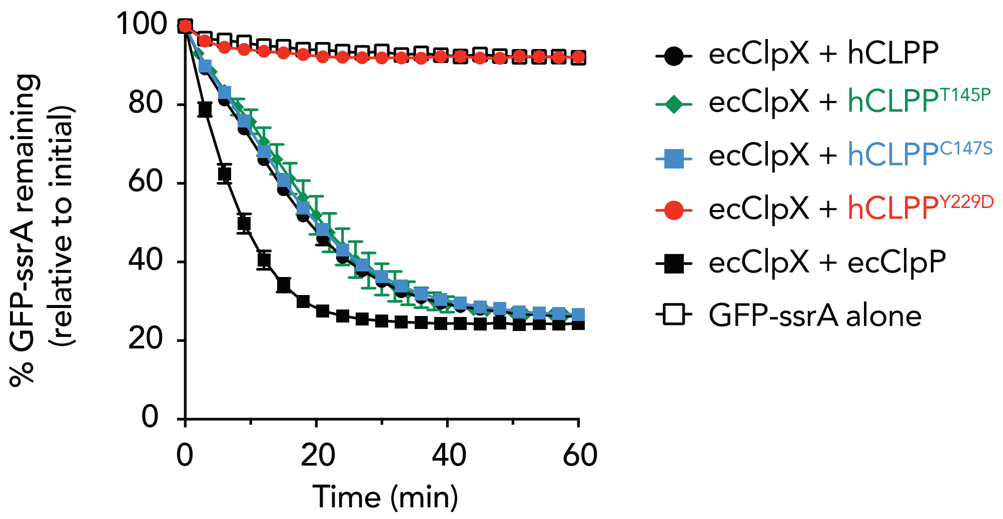

Figure S5 Brodie et al.,

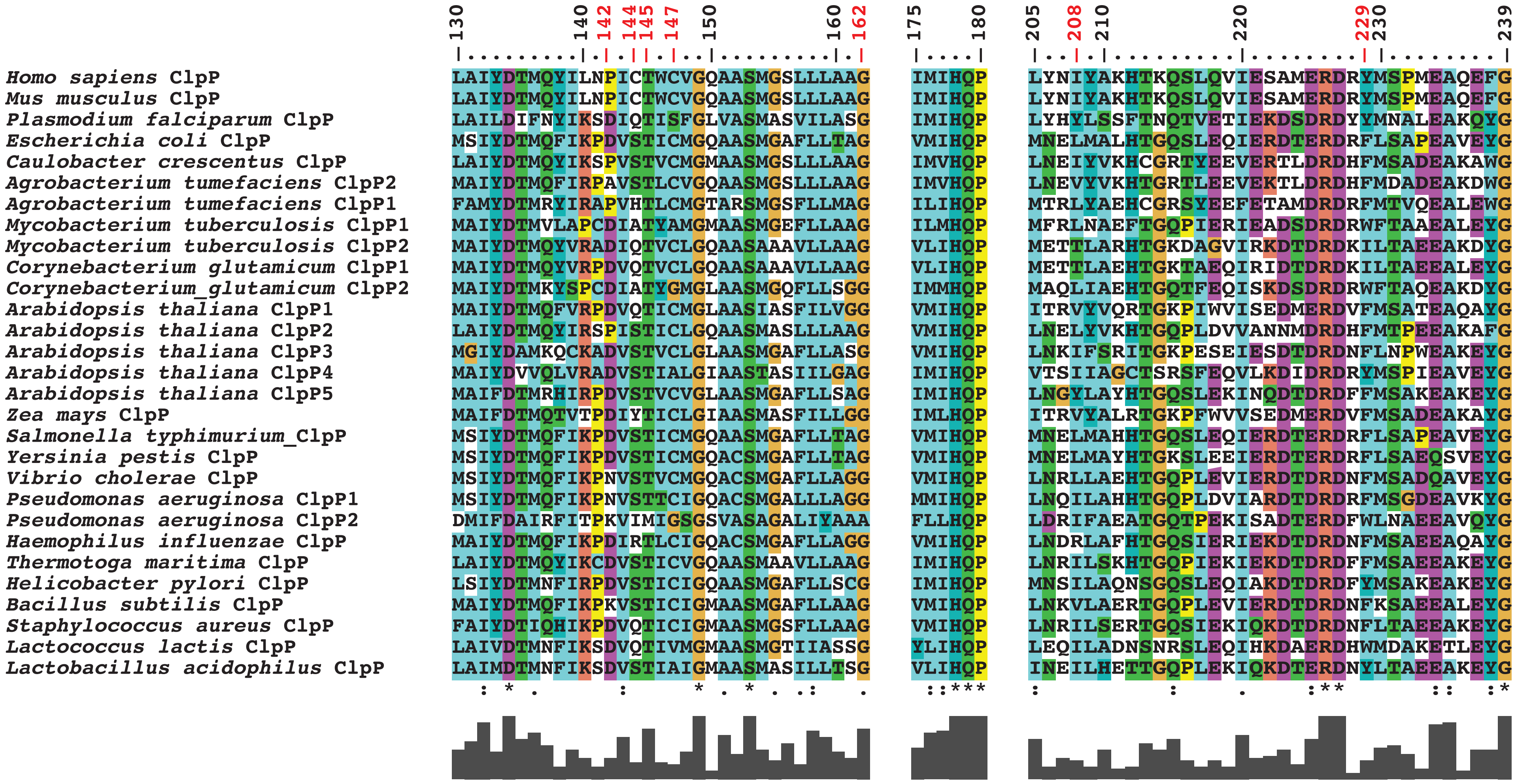

Brodie et al., Figure S6

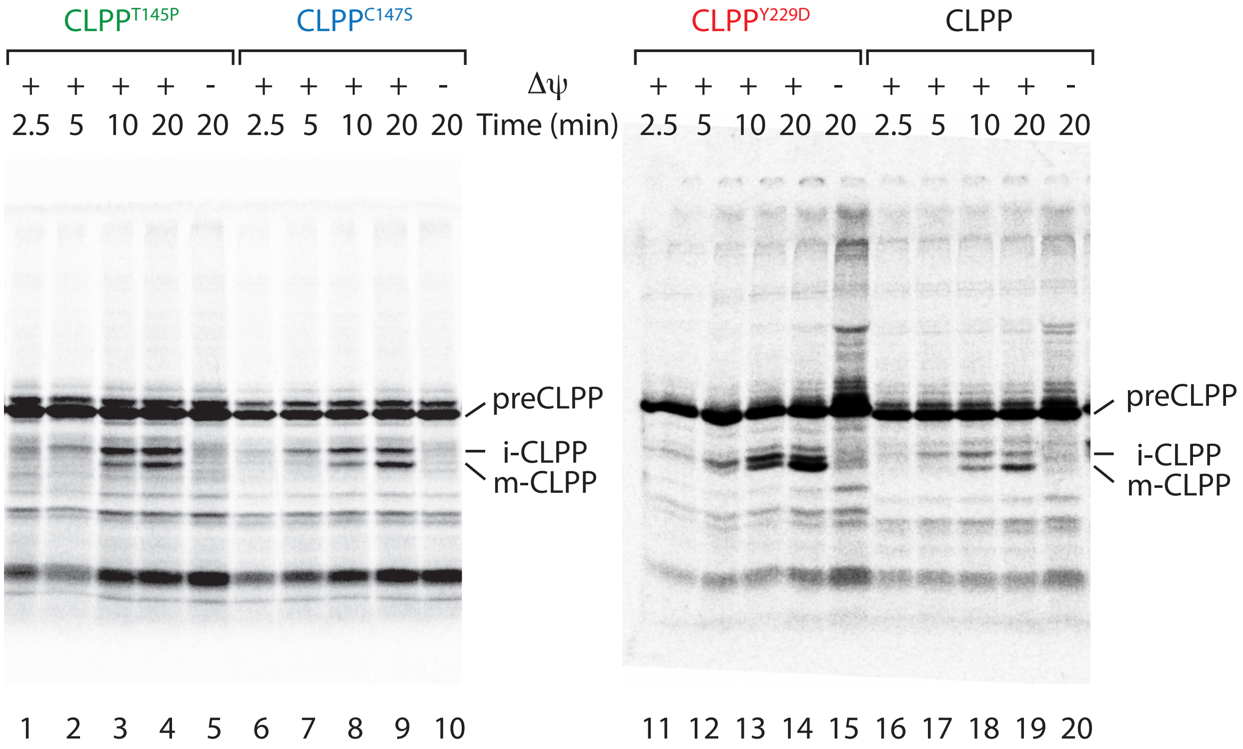

Figure S7 Brodie et al.,

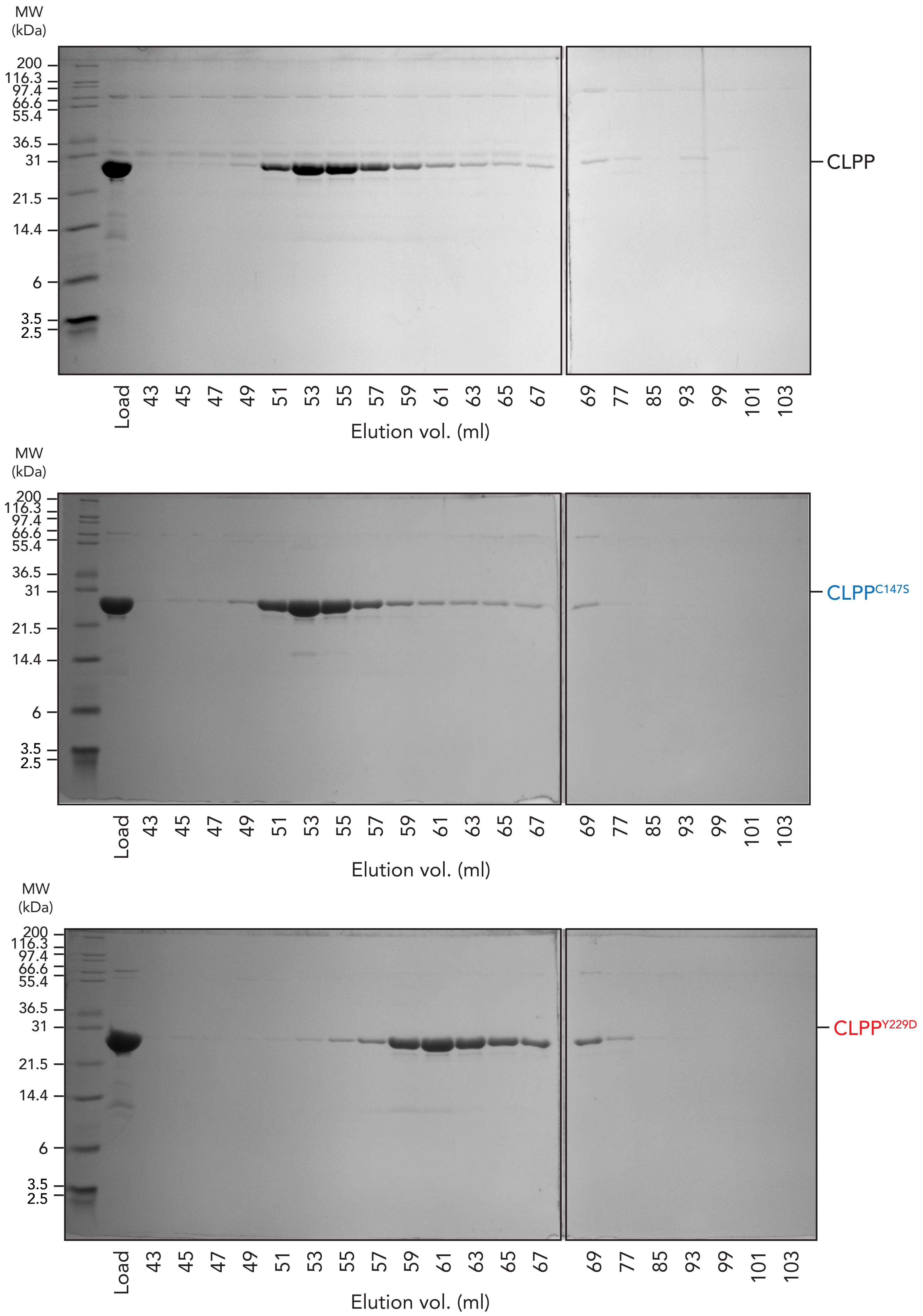

Figure S8 Brodie et al.,

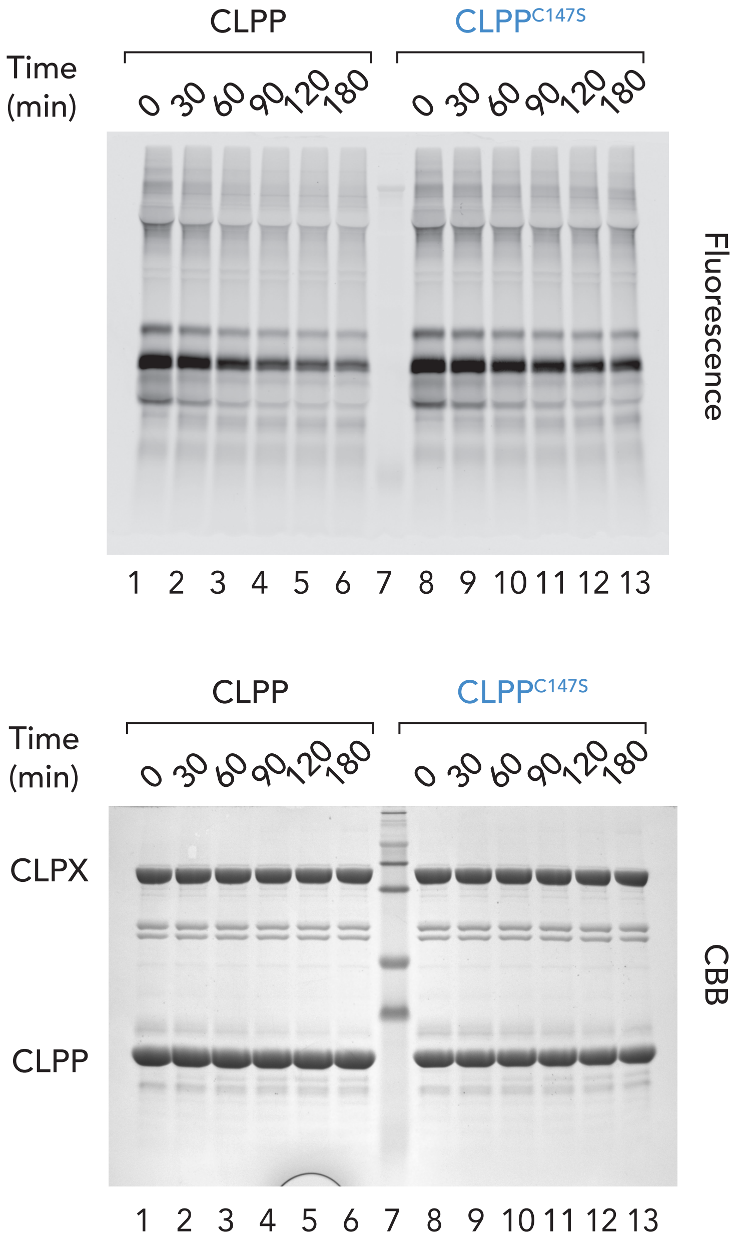

Figure S9 Brodie et al.,

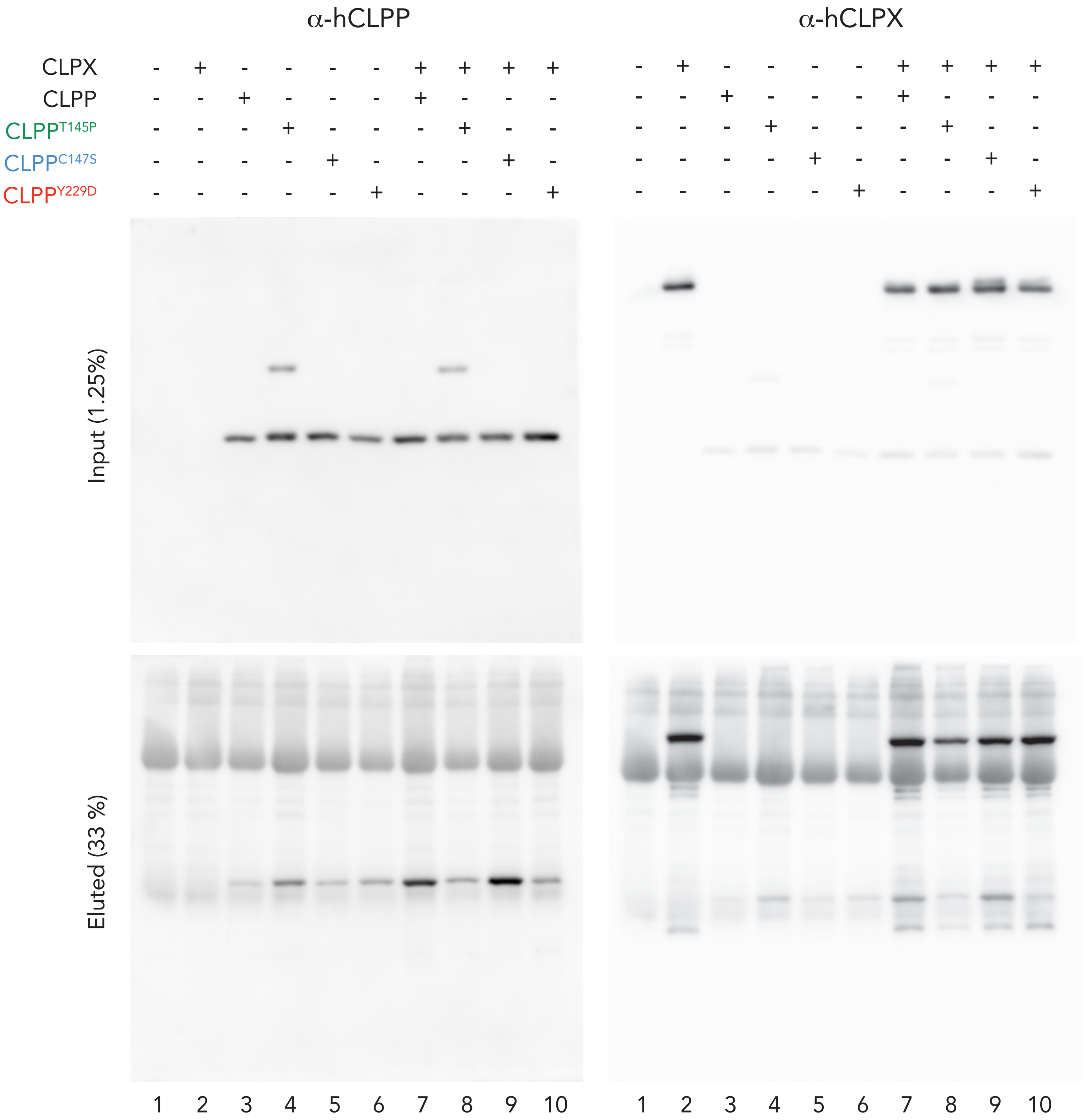

Figure S10 Brodie et al.,
